# Waterborne Cross-Linkable Polyacrylate Latex Coatings with Good Water Resistance and Strength Stabilized by Modified Hectorite

**DOI:** 10.3390/polym13152470

**Published:** 2021-07-27

**Authors:** Yunfan Yang, Jinyang Chen, Guoli Ma, Dingqing Yang

**Affiliations:** Chemical Engineering and Technology, Shanghai University, Shanghai 200444, China; madelia@shu.edu.cn (G.M.); ydq@shu.edu.cn (D.Y.)

**Keywords:** polyacrylate latex films, Pickering emulsion, copolymer, coatings, surface modification

## Abstract

Polyacrylate emulsions were prepared by Pickering emulsion polymerization with multi-modified hectorite as a modifier. The proper wettability of modified hectorite and the stability of O/W emulsions prove that modified hectorite has good emulsification properties. The stability of polyacrylate latexes and the morphology of polymer latex particles were then investigated to explain the role of multi-modified hectorite in stabilizing polyacrylate latex. In addition, the improved mechanical properties and water resistance of the latex make it a potentially excellent coating. Multi-modified hectorite as an alternative modifier to conventional surfactants offers a potential application of nanosolid particles that meet the partial wetting conditions for water and oil as stabilizers for the production of latexes for coatings.

## 1. Introduction

Polyacrylate latex is prepared by emulsion polymerization, which provides an environmentally friendly way to produce emulsion polymers for coatings due to its use of water as a dispersion medium rather than traditional organic solvents [1]. However, polyacrylate latexes usually use surfactant emulsifiers in the preparation process. Small molecule surfactants adhere to the surface of the emulsion droplets by physical adsorption and are easily desorbed by external interference, leading to collisions and the agglomeration of emulsion droplets [2]. In addition, this also reduces the water resistance of the emulsion curing film because of its migration to the surface of film [3].

To solve this issue, surfactant-free emulsion polymerization could be applied. This new method of emulsion polymerization has been developed in previous research. For example, Abdollahi et al. [4] utilized a one-step emulsifier-free emulsion polymerization strategy to synthesize functionalized latex nanoparticles with different morphologies, Cao et al. [5] used graphitic carbon nitride as an initiator and stabilizer for surfactant-free emulsion photo-polymerization to synthesis polymer latexes, and Huang et al. [6] employed reverse iodine transfer polymerization to prepare emulsifier-free acrylate-based emulsion without any addition of surfactant.

Pickering emulsion polymerization is another method of surfactant-free emulsion polymerization. For Pickering emulsions, solid particles with moderate wettability are irreversibly adsorbed at the oil–water interface, which reduces the interfacial energy and provides a physical barrier to prevent the coalescence of the dispersed phase [2,7]. Pickering emulsions produce latex polymers that are as stable as classical polyacrylate emulsions stabilized by conventional surfactants. The stability of Pickering emulsions depends on the total stress of the particles in the liquid and the energy required to remove the particles from the oil–water interface [8].

**Scheme 1:**
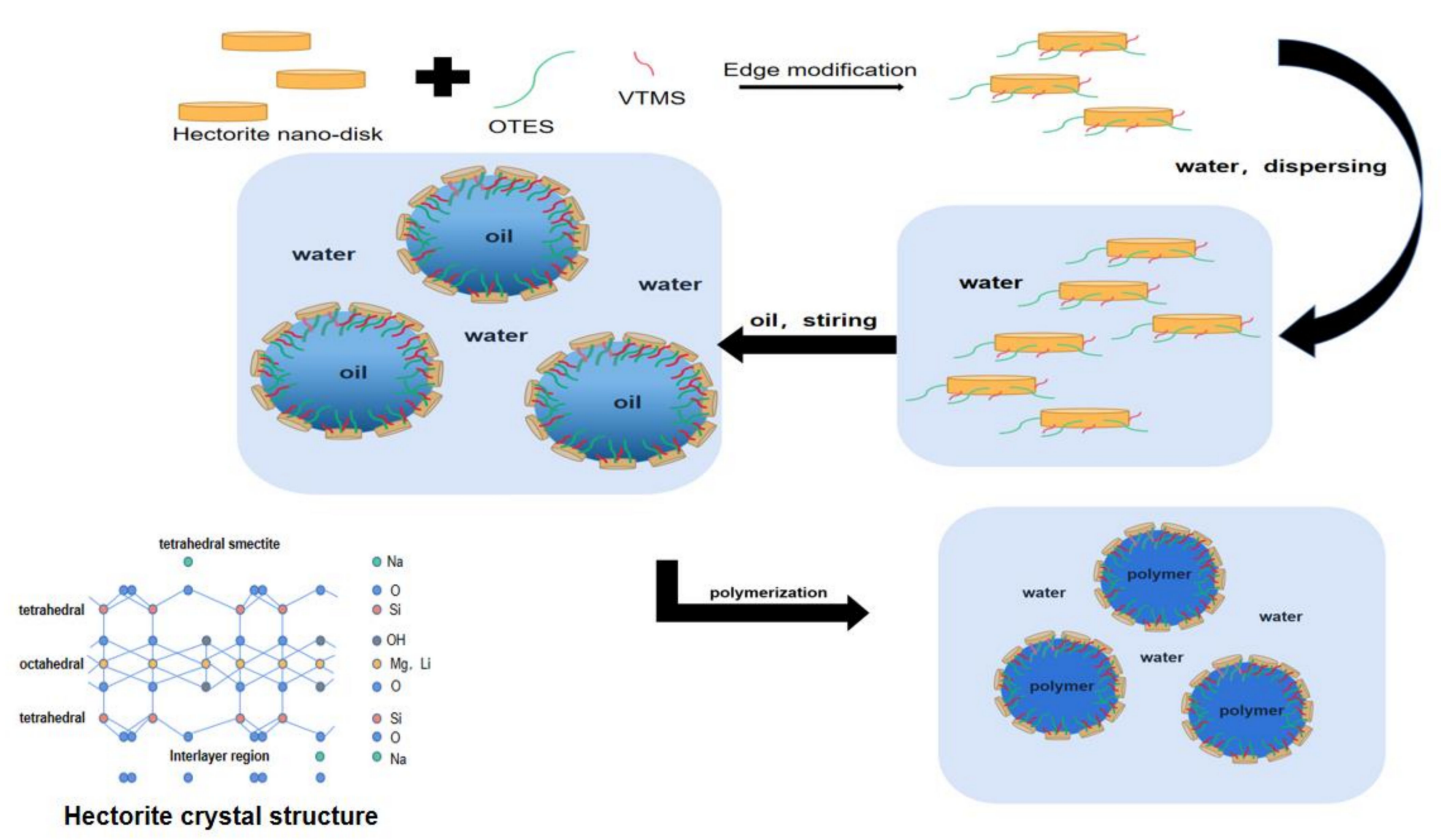
Illustration for preparation of edge-modified amphiphilic hectorite and Pickering emulsions.

There are several particles that meet the partial wetting conditions for water and oil that can be used as Pickering emulsion emulsifiers, including calcium carbonate [6], clays (montmorillonite and hectorite) [9,10,11,12,13], magnetic particles [14], carbon nanotubes [15], and carbon black [16]. Hectorite is a montmorillonite-like nanoparticle with oppositely charged surfaces and edges (50–55 mEq/100 g negative charge density on the surface and 4–5 mEq/100 g positive charge density on the edge). Hectorite, whose structure is shown in Scheme 1, has a nano-disk shape (25 nm in diameter and 1 nm in thickness) that can be modified at the edges because hydroxyl groups are present only at the edge [17]. Thus, the hydrophilic hectorite can be modified so that its edges and surfaces have opposite properties, which makes it a viable emulsifier for Pickering emulsions.

Our work is shown in Scheme 1, where hectorite nano-discs are combined with n-octadecyltrimethoxysilane (OTES) and vinyltrimethoxysilane (VTMS), and then long chain alkyl, vinyl, and siloxyl groups are attached to the edges of hectorite to make the surface amphiphilic. When the hydrophilic surface is tightly attached to the water phase, the hydrophobic molecules reach into the oil phase like tree roots. In addition, the vinyl group can be copolymerized with acrylate monomers, which further stabilize the nano-discs at the oil–water interface, leading to the stability of the emulsion. In addition, siloxyl groups can play an important role in the film-formation process.

## 2. Materials and Methods

### 2.1. Materials

Methyl methacrylate (MMA, ≥98.0%), butyl acrylate (BA, ≥98.0%), and acrylic acid (AA, 99.0%), as the acrylate monomers, were all purchased from Sigma Aldrich Chemical Reagent Co., Ltd. (Shanghai, China). Potassium persulfate (K_2_S_2_O_8_), sodium bicarbonate (NaHCO_3_), and sodium dodecylsulfate (SDS), used as thermal initiator, buffering agent, and emulsifier, respectively, were purchased from Sinopharm Chemical Reagent Co., Ltd. (Shanghai, China). Hectorite was purchased from Zhejiang Hongfeng New Material Co., Ltd. (Zhejiang, China). The OTES (C_21_H_46_O_3_Si) and VTMS (C_5_H_12_O_3_Si) as modifiers were purchased from Macklin Chemical Reagent Co., Ltd. (Shanghai, China). Toluene as solvent was obtained from Aladdin Biochemical Technology Co., Ltd. (Shanghai, China). Deionized water was used throughout the experiment.

### 2.2. Modification of Hectorite

The dosage formulations of hectorite, OTES, and VTMS are given in Table 1. The modification process was as follows: before the reaction, hectorite was dried under vacuum at 60 °C for 12 h. Subsequently, hectorite, OTES, and toluene (150 mL) were mixed in a 250 mL flask and stirred at ambient temperature for 5 days. The product was separated by centrifugation (500× rpm for 3 min), further washed with toluene to remove excess OTES, and dried under vacuum at 40 °C for 24 h. Finally, the product was washed with toluene at least three times to remove excess VTMS and further dried under vacuum at 60 °C.

### 2.3. Preparation of O/W Emulsions and Synthesis of Polyacrylate Latexes

The hectorite product was dispersed into 80 mL of deionized water and sonicated at 330 W for 30 min using an ultrasonic crusher to form the aqueous phase. After that, an oil phase consisting of 5 g MMA, 5 g BA, and 0.25 g AA was prepared. The amount of hectorite was 3% of the total mass of the monomers. The obtained aqueous and oil phases were mixed into a beaker and pre-emulsified using FM200 high shear dispersion emulsifier (Youyi Instrument Co., Ltd., Shanghai, China) at 15,000× rpm for 20 min to obtain an oil–water emulsion.

Polyacrylate latex was prepared by the Pickering emulsion polymerization method. A quantity of 30.0 g of oil/water emulsion was added to a 250 mL flask, then 2 mL of aqueous NaHCO_3_ solution (0.1 g/mL) was added to the system, the temperature was raised to 80 °C, and 4 mL of aqueous KPS solution (0.05 g/mL) was added to initiate the reaction. The reaction was held for a period of time and the remaining emulsion was added. Finally, the pre-emulsion was cast half an hour after the end of the holding period of 1 h to obtain polyacrylate latex.

### 2.4. Characterization

FTIR spectra were obtained by an FTIR spectrophotometer (Vertex 70, Bruker, Karlsruhe, Germany) from 400 to 4500 cm^−1^. Thermogravimetric analysis (TGA5500, Perkin Elmer, Waltham, MA, USA) was performed by the weight change of the sample from 30 to 800 °C under a nitrogen atmosphere with a heating ratio of 20 °C/min. The contact angle analyzer (CA, JC2000C1, Zhongchen Powereach Company, Shanghai, China) was used to measure the water contact angle (WCA) of nanoparticles. At least five different locations were selected on the surface of the hectorite nano-powder sheet and deionized water was dropped. When the water drops stopped moving, static contact angle pictures were recorded.

Images of the oil/water emulsion were taken using an optical microscope (BX63, Olympus, Tokyo, Japan). The mean droplet diameter and size distributions of oil/water emulsions based on optical images were analyzed by Image-Pro software. The morphology of the polyacrylate emulsions prepared by Pickering emulsion polymerization was observed with a TEM (Tecnai F20, FEI Corp, Waltham, Massachusetts, USA) at 200 kV. Prior to observation, the samples were stained with phosphotungstic acid (1 wt%). The particle size and size distributions of the prepared polyacrylate latexes were measured at room temperature using a Nano-Zetasizer instrument (Malvern Zetasizer Nano ZS90, Malvern, Worcestershire, UK). The dispersion was diluted to approximately 1 wt% with distilled water before starting the measurements. Three sets of experiments were conducted for each latex sample and the average value was taken.

The coating was cast on a 25 × 75 mm^2^ glass sheet with a tie rod at ambient temperature to obtain a wet film thickness of 200μm. The mechanical properties of the films were performed on a tensile testing machine (CMT41-4, Xie Qiang Instrument Manufacturing Co., Ltd., Shanghai, China) with a stretching speed of 100 mm/min. The water contact angles of these films were measured under the same conditions described above.

### 2.5. Swelling Test

The film was first weighed as m_1_ and then placed in a flask with trichloromethane as a solvent until equilibrium was reached. The swollen film was wiped with filter paper and weighed as *m*_2_. The swelling ratio was calculated by (*m*_2_/*m*_1_) × 100%. After drying in a heating oven under vacuum at 60 °C for 48 h, the dried film was weighed as m_3_. The crosslinking degree was calculated by (*m*_3_/*m*_1_) × 100% [18].

### 2.6. Water Absorption Test

A latex film was taken and cut into 10 × 10 mm^2^ samples and the initial film mass weighed on an electronic balance and recorded as m_0_, before being completely immersed in distilled water. After that, the sample was weighed periodically on the analytical balance as *m*_1_. The water absorption of the film was calculated by (*m*_1_ − *m*_0_)/*m*_0_ × 100% [19].

## 3. Results and Discussion

### 3.1. Modification of Hectorite

**Scheme 2:**
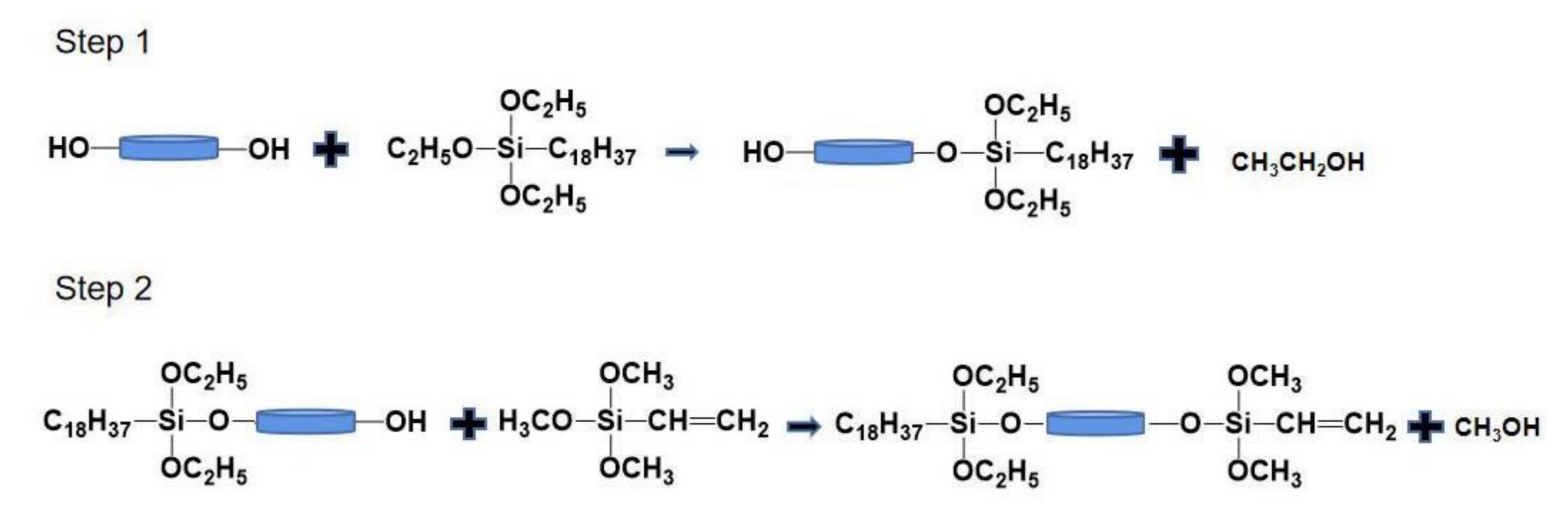
Schematic illustration of the grafting of OTES and VTES onto nano-disk hectorite.

The modification process is divided into two steps as shown in Scheme 2. Step one is achieved by a reaction between the ethoxy group of OTES and the hydroxyl group on the edge of the hectorite, with the release of ethanol molecules. Step two has a similar reaction mechanism, in which the methanol molecules are stripped and then the alkyl chains and vinyl and siloxy groups are attached to the hectorite.

**Figure 1:**
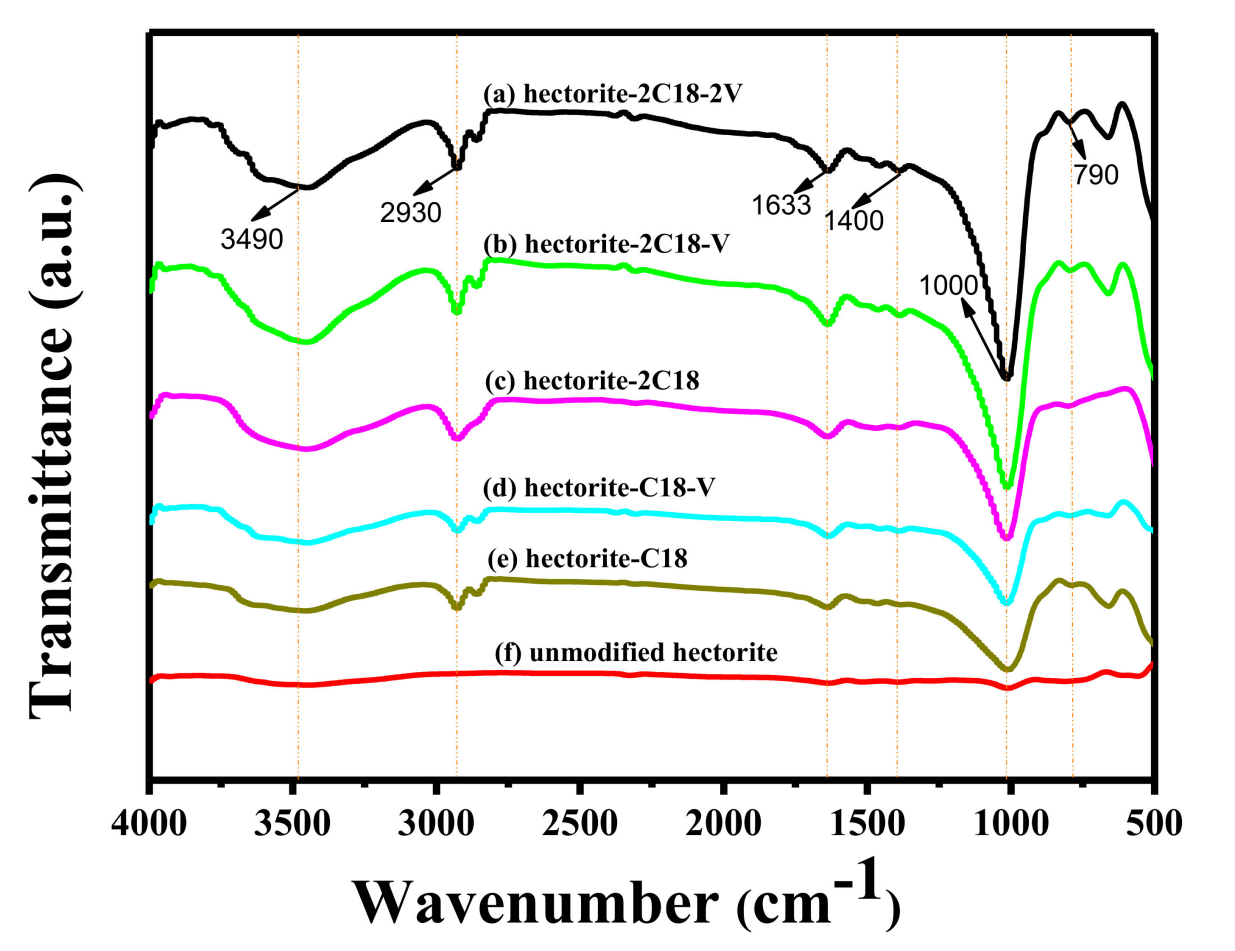
FTIR spectra of unmodified and modified hectorite prepared with different OTES/VTMS content.

The successful modification of hectorite was confirmed by Fourier transform infrared spectroscopy (FTIR) and thermal gravimetric analysis (TGA). The FTIR spectra of unmodified and modified hectorite shown in Figure 1 give qualitative evidence of alkane chains chemically anchored at the hectorite edge. The characteristic absorption peaks at 1000 and 3490 cm^−1^ originated from H-OH and the stretching vibration of Si–O; the bending vibration peak of Si–OH was detected at 1633 cm^−1^. Compared with unmodified hectorite, the characteristic absorption peak at 2930 and 790 cm^−1^ was ascribed to the stretching vibration of CH_3_ and CH_2_, revealing that the hydroxyl group of hectorite reacted with the siloxy groups of OTES and that the alkyl chain was grafted on the hectorite. The stretching vibration absorption peak of the C=C which appeared at 1633 cm^−1^, when compared to unmodified hectorite and hectorite modified with OTES only, also proved the successful graft of the vinyl group from VTMS on the hectorite.

**Figure 2:**
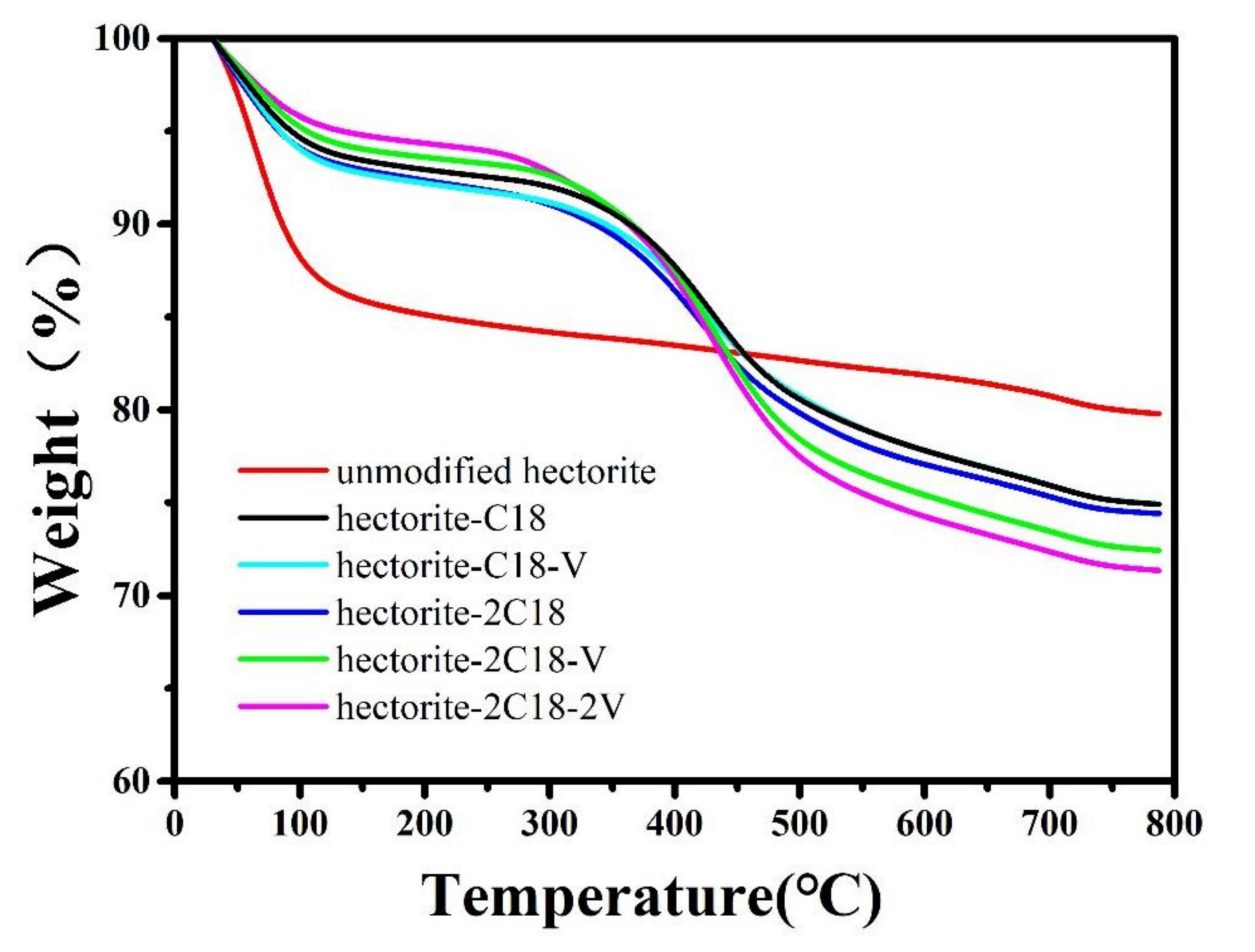
TGA degradation profiles of unmodified and modified hectorite prepared with different OTES/VTMS content.

The TGA curves of the modified and unmodified hectorite presented in Figure 2 give quantitative evidence supporting the conclusion based on FTIR. The weight loss between 200 and 600 °C due to the thermal decomposition of the organic molecule was considered, while the region below 200 °C, which could be ascribed to the removal of adsorbed water, is excluded. Compared with the curve of unmodified hectorite, the curve of modified hectorite has two stages of weight loss, demonstrating the hydroponic chains on the hectorite. The number of hydroponic chains grafted to the hectorite could be estimated by Equation (1) [10]:

(1) $$\mathrm{Grafted}\;\mathrm{amount}=\frac{10^{3}\left(W_{modified\;hectorite}-W_{hectorite}\right)}{[100-(W_{modified\;hectorite}-W_{hectorite)]M}}$$

where *M* (g/mol) is the molecular weight of the grafted molecules. The grafted amount was estimated as 8.6, 9.1, 9.3, 10.1, and 10.7 wt% for hectorite-C18, hectorite-C18-V, hectorite-2C18, hectorite-2C18-V, and hectorite-2C18-2V samples, respectively, which corresponds to the amount of modifier given in Table 1. The results are consistent with the rule that the more modifier is added, the more hydrophobic groups are grafted at the hectorite.

**Figure 3:**
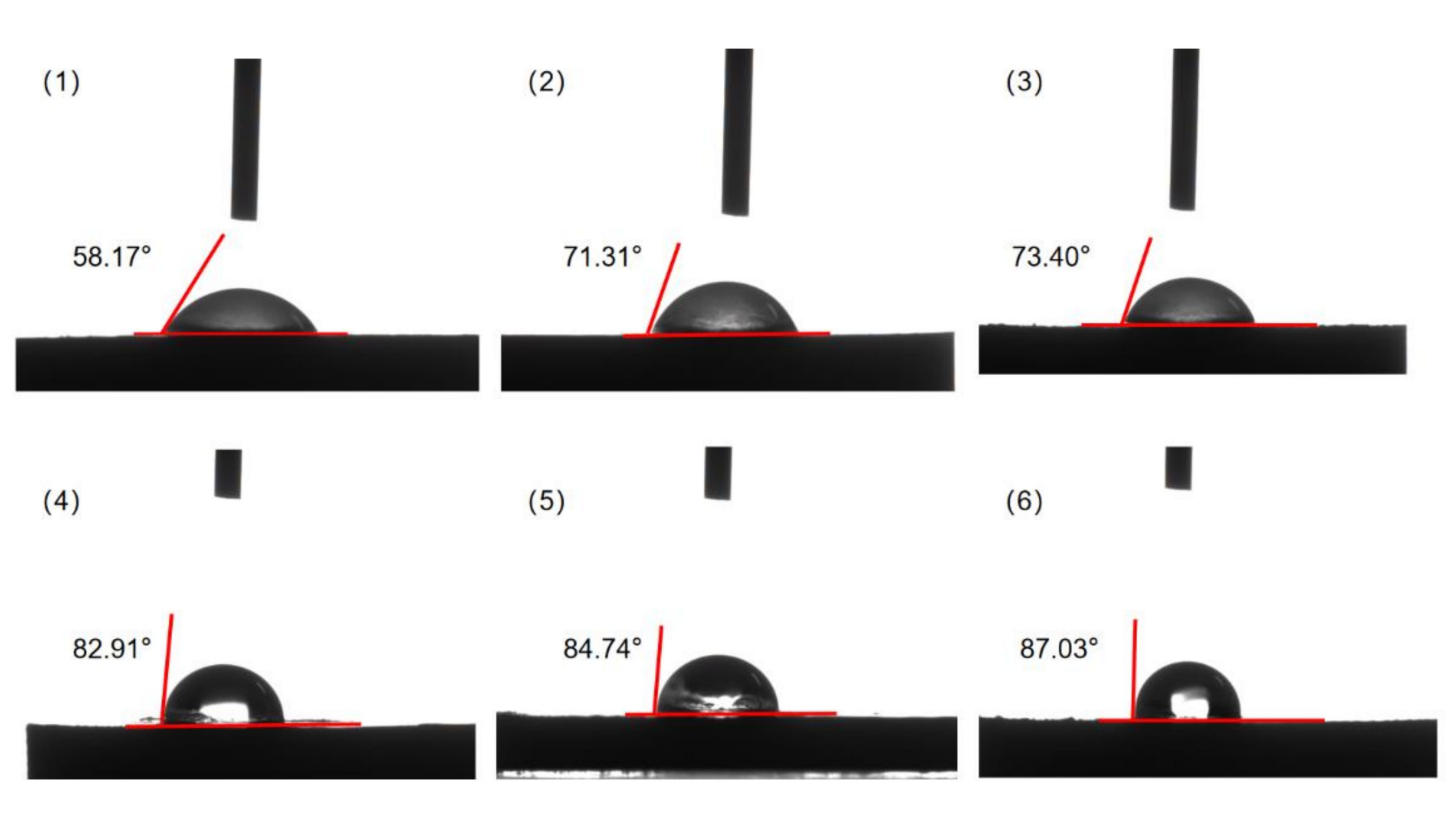
Photographs of contact angle of (**1**) unmodified hectorite, (**2**) hectorite-C18, (**3**) hectorite-C18-V, (**4**) hectorite-2C18, (**5**) hectorite-2C18-V, (**6**) hectorite-2C18-2V.

Adsorption energy plays the most important role in the stability of a Pickering emulsion and determines whether solid particles can stably stay at the interface. It is known that contact angle, particle size, and surface tension are the factors that affect adsorption energy. The energy(E) required to remove a particle of radius d from an oil–water interface of tension *γ_ow_* is given by Equation (2) [2]:

(2) $$E=\pi {\left(\frac{d}{2}\right)}^{2}\gamma_{ow}{\left(1\pm cos\theta \right)}^{2}$$

where the sign is positive for the move into the oil phase, and negative for the move into the water phase. According to Equation (2), the closer the contact angle θ is to 90°, the higher the energy and the better the stability of the Pickering emulsion [2,20,21]. Figure 3 is the photographs of the contact angle and it shows that with the increase of the amount of modifier, the water contact angle of the modified hectorite gradually increased and became closer to 90° than that of the unmodified hectorite. By analogy with surfactant molecules, particles whose contact angle is below 90° lead to a larger fraction of the particle surface residing in water than in oil, and give rise to O/W emulsions. Thus, the O/W Pickering emulsion of polyacrylate could be produced by the modified hectorite. Contrasting the different variations of contact angles revealed that OTES is much more efficient for improving the wettability of hectorite than VTMS, which could be due to the excellent hydrophobic properties of long-chain alkanes. In addition, the incremental hydrophobicity of the modified hectorite also proves that the modification procedures work.

### 3.2. Pickering Emulsion Stabilized by Hectorite

**Figure 4:**
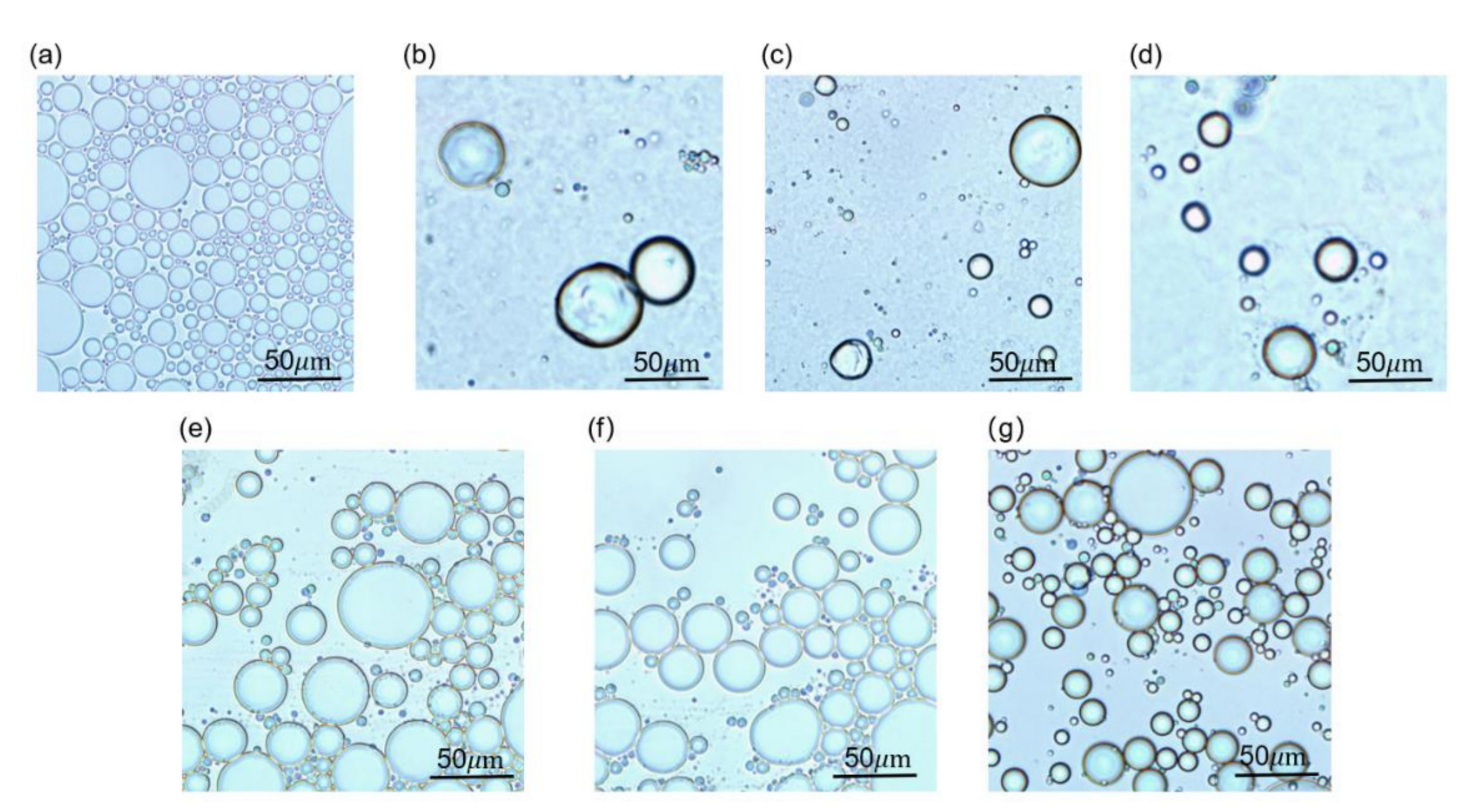
Microscopy images of emulsions emulsified by (**a**) SDS, (**b**) unmodified hectorite, (**c**) hectorite-C18, (**d**) hectorite-C18-V, (**e**) hectorite-2C18, (**f**) hectorite-2C18-V, (**g**) hectorite-2C18-2V were captured after the as-obtained emulsions were stored for 24 h.

O/W emulsions with 3 wt% of unmodified hectorite, hectorite-C18, hectorite-C18-V, hectorite-C18-2V, hectorite-2C18-V, and hectorite-2C18-2V as emulsifier were prepared, and their emulsifying properties were compared with those of traditional emulsifier SDS (2 wt%). After 24 h of storage, it could be observed that the O/W emulsions emulsified by unmodified hectorite, hectorite-C18, and hectorite-C18-V were stratified while other emulsions were stable. Microscopy images of emulsions emulsified by different stabilizers are shown in Figure 4, and an average value of droplet diameter adopted from the individual droplets is shown in Table 2. The O/W emulsions emulsified by unmodified hectorite, hectorite-C18, and hectorite-C18-V had larger droplet sizes and much smaller quantities, which indicates the non-ideal emulsification properties of these particles. It could be inferred that a hectorite nano-disk with high hydrophilicity cannot play the role of stabilizing O/W emulsions because the lower adsorption energy makes the hydrophilic particles tend to aggregate in the aqueous phase rather than at the oil–water interface.

It was observed that the emulsion emulsified by hectorite-2C18-2V showed the smallest droplet size distribution and the highest number of droplets. The droplet size distribution decreased and the droplet number increased in the O/W emulsion stabilized by hectorite-2C18-V, while these properties observed in the emulsion emulsified by hectorite-2C18 were the worst of the three, which is consistent with the adsorption energy theory [2]. Furthermore, compared with traditional surfactant SDS, hectorite-2C18-2V also showed similar emulsifying properties. It is worth mentioning that no stratification occurred in emulsions emulsified by hectorite-2C18, hectorite-2C18-V, and hectorite-2C18-2V after 1 month of storage, indicating that amphiphilic solid particles could be adsorbed at the O/W interface to form a physical barrier against coalescence, which would endow it with excellent emulsification performance.

Polyacrylate latex stabilized by hectorite was prepared. After the polymerization reaction, the unmodified hectorite, hectorite-C18, and hectorite-C18-V could not produce a stable latex and a significant aggregation of polymer particles resulted in the formation of coarse polymer clumps. As for formulations including hectorite-2C18, hectorite-2C18-V, and hectorite-2C18-2V, they were also difficult to obtain stable latex from when the particle content was less than 3 wt%, while a stable milky latex without coagulum was produced when the particle content was more than 3 wt%.

The polyacrylate latex stabilized by hectorite-2C18, hectorite-2C18-V, and hectorite-2C18-2V remained stable after a period of storage of more than 3 months, indicating that appropriate wettability and adequate mass fraction are necessary for the preparation of stable Pickering polyacrylate emulsion. As for stability, thermodynamically stable Pickering emulsion systems are superior to kinetically stable polyacrylate emulsion systems stabilized by SDS because of the irreversible adsorption capacity of modified hectorite at the oil–water interface [2].

**Figure 5:**
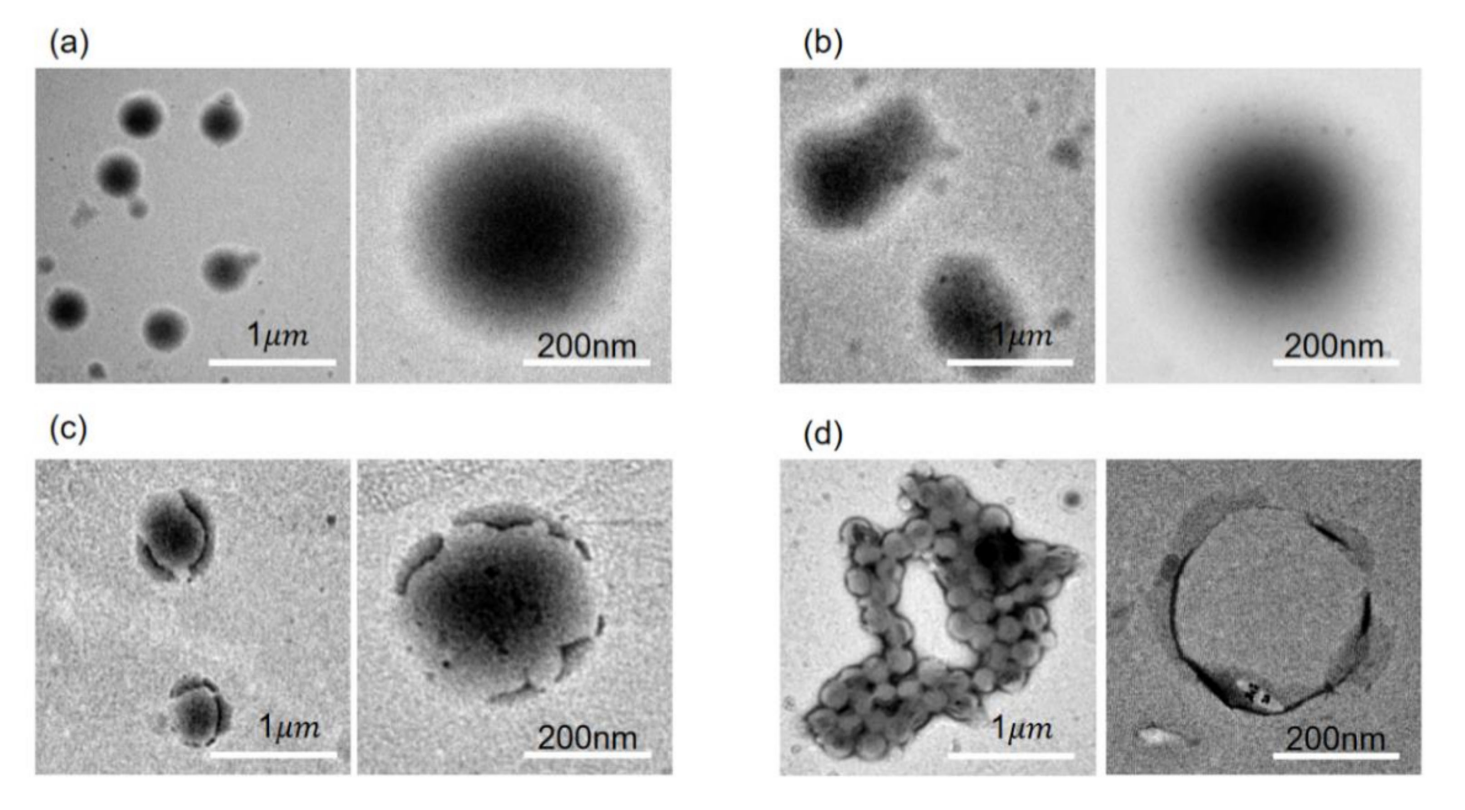
TEM images of polyacrylate latex stabilized by different stabilizers: (**a**) SDS, (**b**) hectorite-2C18, (**c**) hectorite-2C18-V, (**d**) hectorite-2C18-2V.

**Figure 6:**
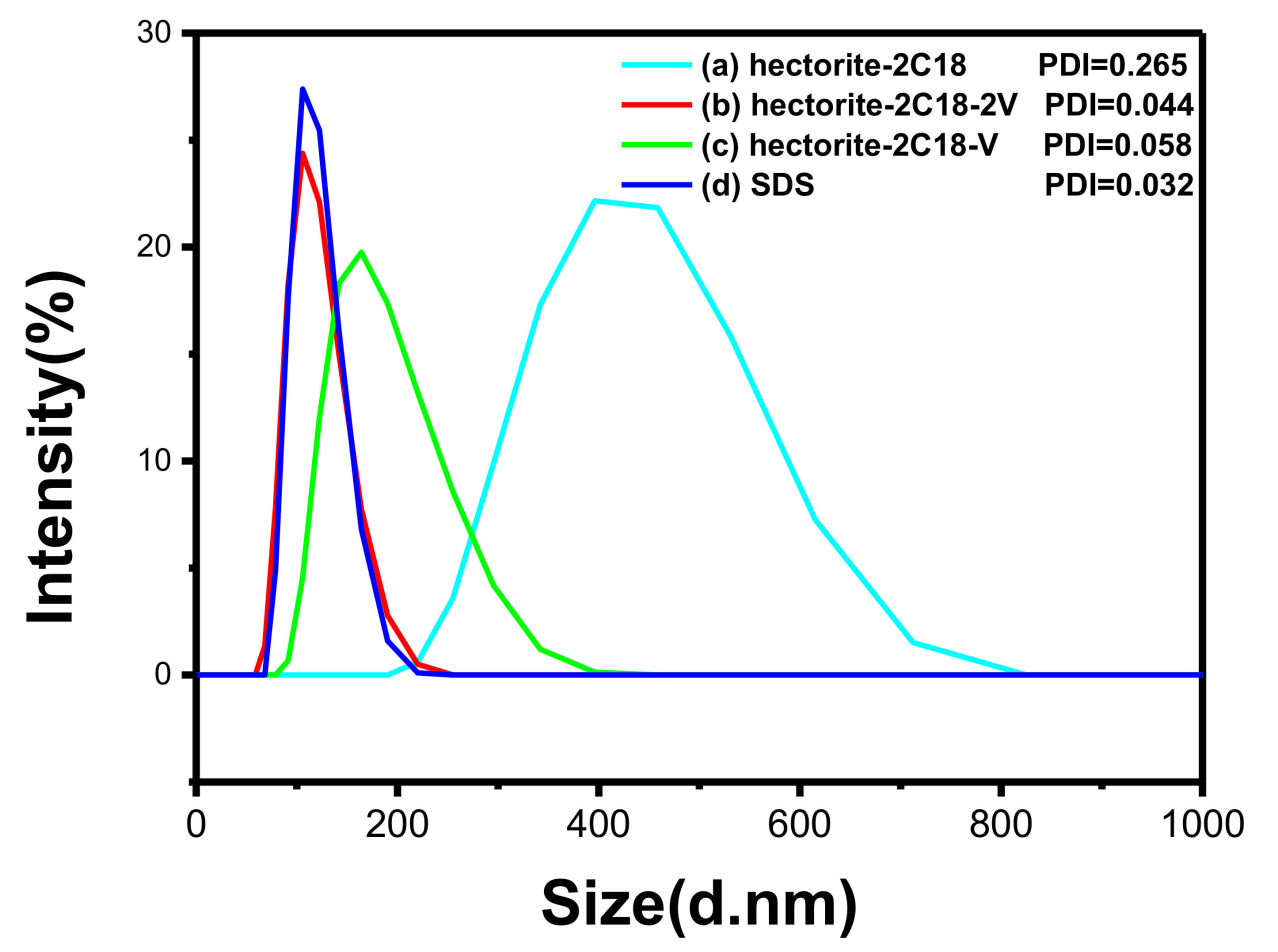
DLS results of polyacrylate latex stabilized by different stabilizers: (**a**) stabilized by hectorite-2C18, (**b**) stabilized by hectorite-2C18-2V, (**c**) stabilized by hectorite-2C18-V,(**d**) stabilized by conventional surfactant SDS. The dispersion was diluted to approximately 1wt% with distilled water.

The morphology and size of polyacrylate latex stabilized by 1 wt% SDS and 3 wt% modified hectorite (hectorite-2C18, hectorite-2C18-V, and hectorite-2C18-2V) were shown by TEM (Figure 5) and DLS (Figure 6). The polyacrylate latex stabilized by hectorite-2C18 showed an irregular spherical structure with poor dispersion, while the polyacrylate latex stabilized by SDS, hectorite-2C18-V, and hectorite-2C18-2V showed a regular spherical structure with a significantly smaller particle size and good dispersion. Compared with Figure 5b–d, it can be seen that the amount of modified hectorite attached to the surface of latex particles increased significantly and an effective mechanical barrier was formed to against latex coalescence, especially for the polyacrylate latex stabilized by hectorite-2C18-2V. The DLS results are consistent with the particle size shown in the TEM images. As shown in Figure 6, the particle size distribution of polyacrylate latex stabilized by hectorite-2C18-2V is very close to a conventional surfactant SDS stabilized emulsion, indicating that the Pickering polyacrylate latex was comparable to the traditional polyacrylate emulsion stabilized by small molecule surfactants. Furthermore, the polyacrylate emulsions stabilized by hectorite-2C18-V had larger average sizes and wider distributions, while the polyacrylate emulsions stabilized by hectorite-2C18 were much worse.

Based on the above results, the stability mechanism of a Pickering polyacrylate latex should reflect the following pattern. The more suitable wettability of the modified hectorite improved the adhesion and quantity of hectorite at the O/W interface, while the vinyl groups attached to the edge of the modified hectorite further promoted the adhesion of hectorite to the polyacrylate through the copolymerization of vinyl groups with acrylate monomer adhesion of the particles. As a result, the polyacrylate latex was stable under the combined effects of the Pickering effect and covalent bonding. The vinyl played a more important role in the mechanism of stabilizing polyacrylate, because the emulsification performance of multi-modified hectorite (hectorite-2C18-V and hectorite-2C18-2V) was better than that of mono-modified hectorite (hectorite-2C18).

### 3.3. Films Obtained from the Polyacrylate Latexes

#### 3.3.1. Swelling Ratio and Crosslinking Degree

**Figure 7:**
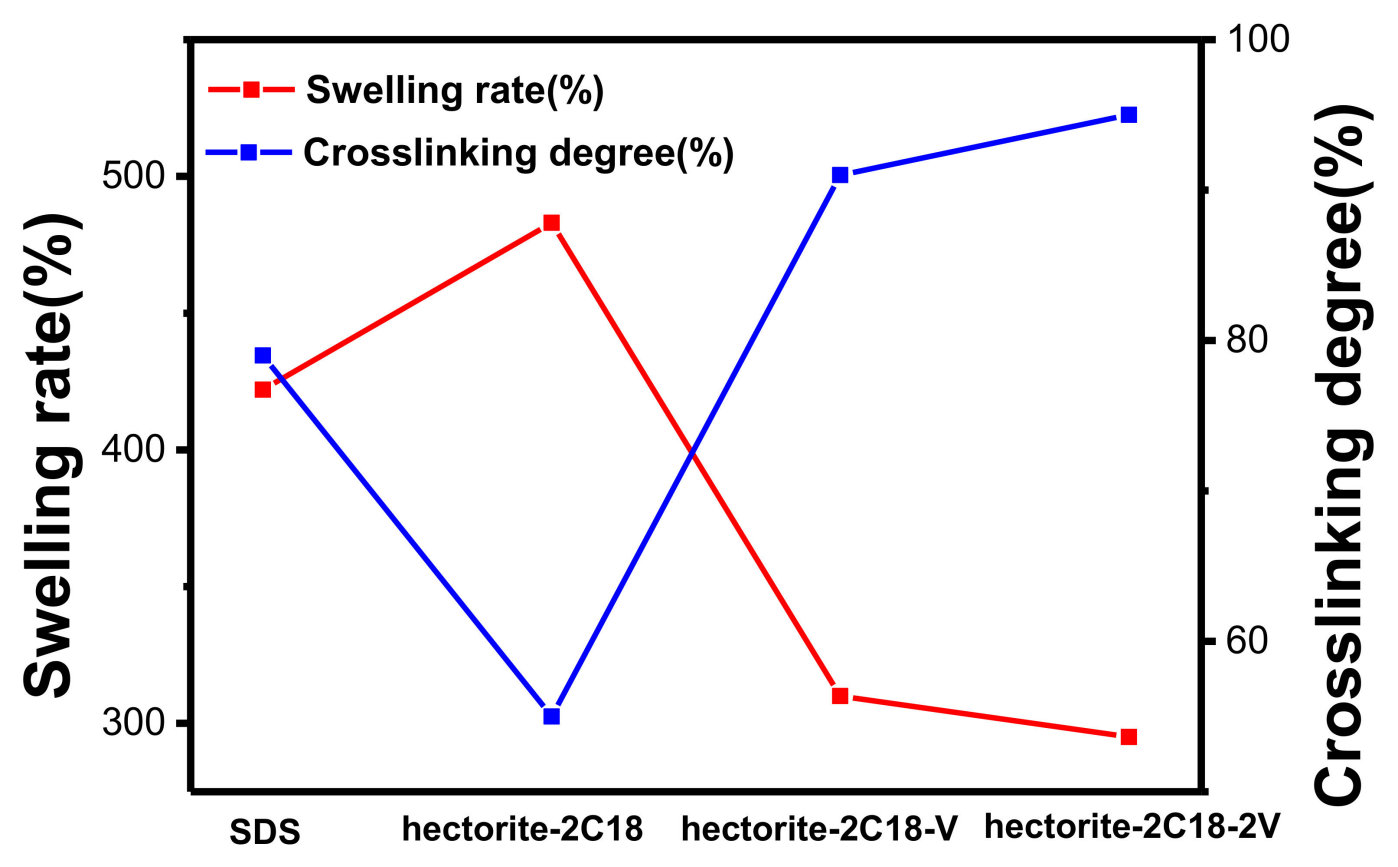
Swelling ratio and crosslinking degree of the cured films from the polyacrylate latex coatings prepared with SDS (1 wt%), Laponte-2C18 (3 wt%), hectorite-2C18-V (3 wt%), hectorite-2C18-2V (3 wt%).

The film usually forms in two stages: (1) With the evaporation of water in the system, the particles are close to each other and the deformation is stacked. (2) A cross-linking curing reaction occurs between the modified hectorite with reactive groups and polymer molecules to form a three-dimensional network structure. As shown in Figure 7, the swelling ratio and cross-linking degree of the cured films was calculated to understand the film-formation of the polyacrylate latex coatings. The swelling ratio was opposite to that of the cross-linking degree with the use of different emulsifiers; the higher the crosslinking degree, the smaller the swelling ratio.

**Scheme 3:**
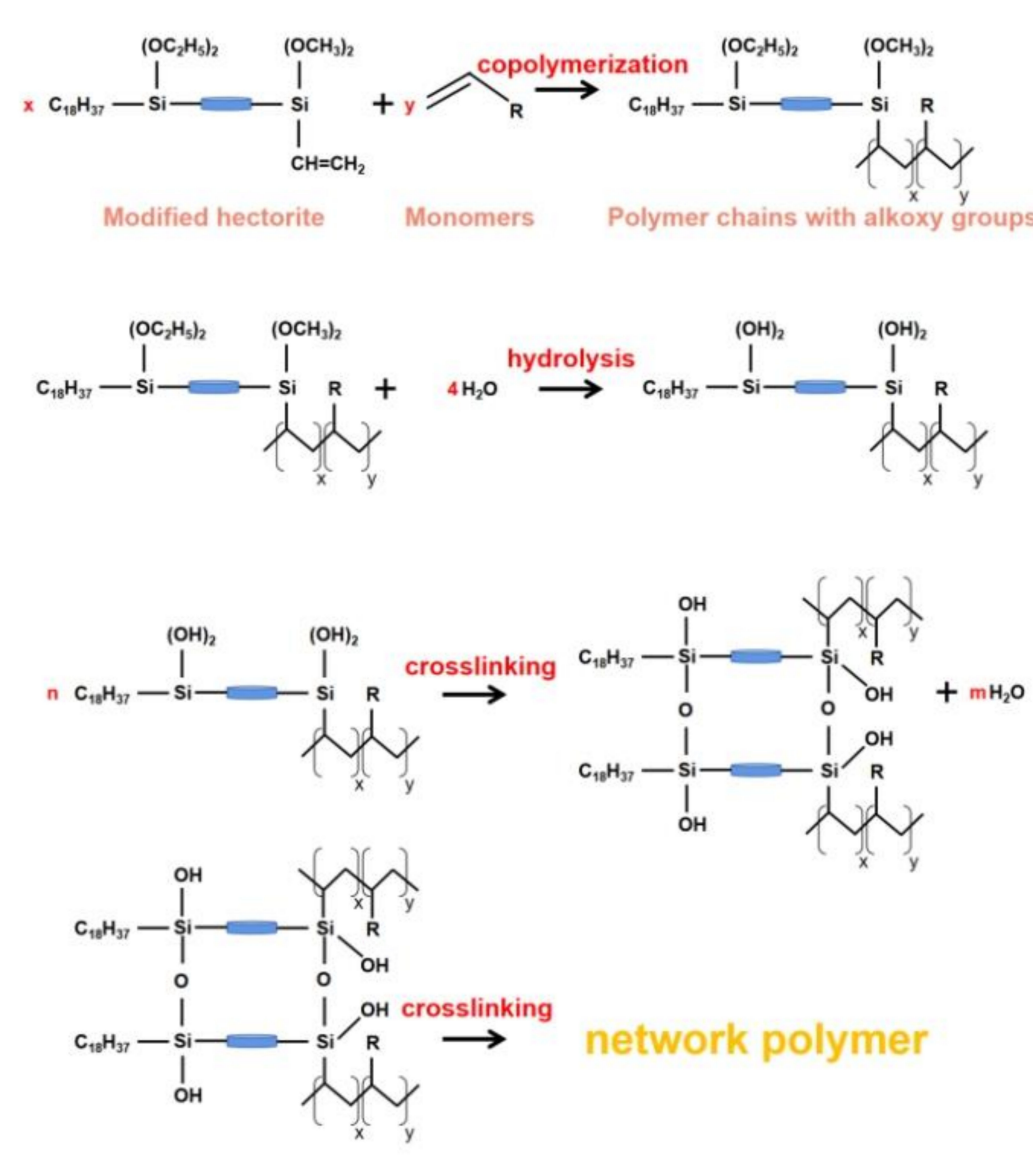
Schematic illustration of the Pickering polyacrylate latex reaction mechanism.

The above results demonstrate that the crosslinking degree of polyacrylate latex stabilized by traditional small molecule surfactant was inferior to that of polyacrylate latex stabilized by hectorite-2C18-V and hectorite-2C18-2V, which could be ascribed to the strong interaction between the hectorite and the polymer molecules because of the reactive vinyl groups around the edge of modified hectorite. In addition, the Si–O–C structures in the organosiloxane on the modified hectorite which had not grafted around the edge went through a hydrolysis reaction, and the crosslinking reaction of the hydroxyl group generated by hydrolysis formed a three-dimensional network structure to increase the crosslinking degree of the emulsion film. The reaction mechanism is shown in Scheme 3.

A comparison of hectorite-2C18, hectorite-2C18-V, and hectorite-2C18-2V revealed that the cross-linking degree of the Pickering polyacrylate latex was related to the size distribution of polymer particles and the number of Si–O–C structures and vinyl groups attached to the stabilizer-modified hectorite. It could be concluded that a smaller size led to the more complete coalescence of polyacrylate droplets, more cross-linkable Si–O–C structures, and vinyl groups that can be covalently linked with polymer molecules to make the cross-linking between polymer molecules more efficient.

#### 3.3.2. Mechanical Strength

**Figure 8:**
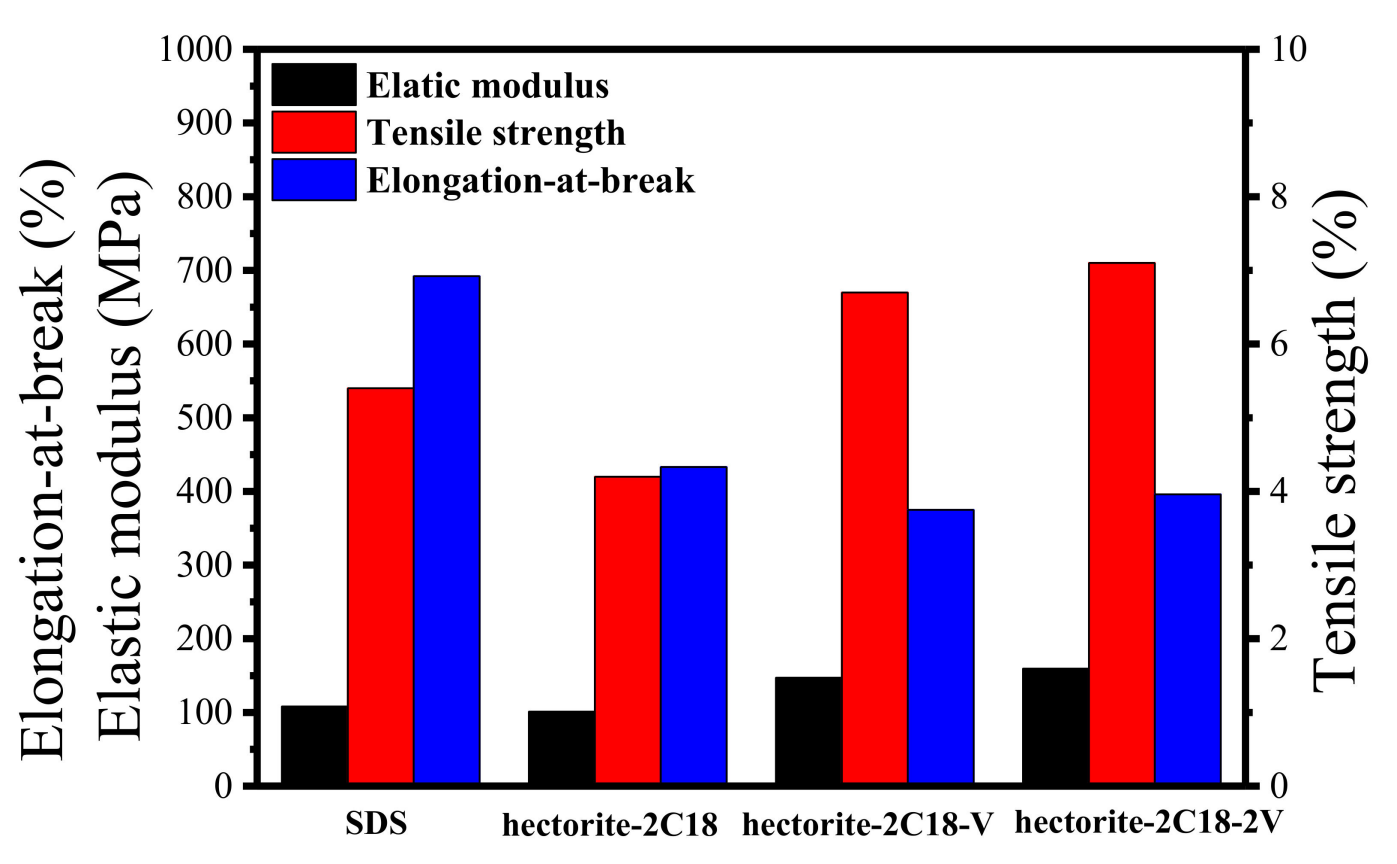
Elastic modulus, tensile strength, and elongation-at-break of polyacrylate latex films prepared with SDS (1 wt%), Laponte-2C18 (3 wt%), hectorite-2C18-V (3 wt%), hectorite-2C18-2V (3 wt%).

The mechanical properties of the material include elastic modulus, tensile strength, and elongation, which are used to measure the elastic deformation resistance, fracture resistance, softness, and elasticity of the film, respectively. As shown in Figure 8, the elastic modulus and tensile strength of polyacrylate films prepared by hectorite-2C18-V and hectorite-2C18-2V were better than those of polyacrylate films prepared by tradition surfactants. The results were consistent with the crosslinking degree as mentioned above, which indicates that a higher cross-linking degree gave the coating better elastic deformation resistance and fracture resistance. On the other hand, it also confirms that the multi-modified hectorite not only improved particle size distribution and the stability of polyacrylate latex, but also played an essential role in forming the three-dimensional crosslinking network as crosslinking points to improve the film’s mechanical properties. In addition, the elongation-at-break presents a trend opposite to the other two mechanical performances; the decreased ratio can be attributed to the fact that the high degree of crosslinking restricted molecular chain motion.

#### 3.3.3. Water Resistance

**Figure 9:**
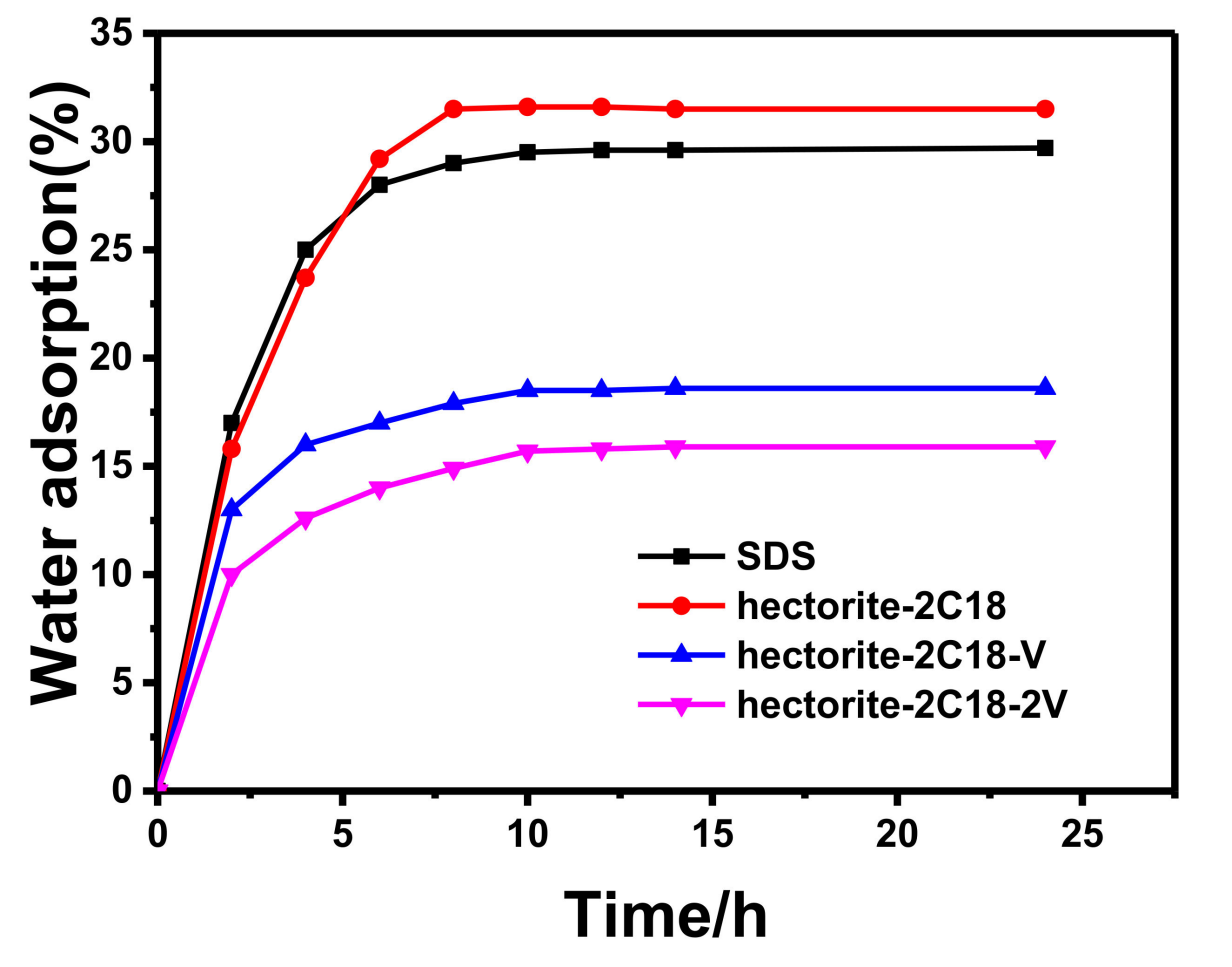
Water adsorption of polyacrylate latex films prepared with SDS (1 wt%), Laponte-2C18 (3 wt%), hectorite-2C18-V (3 wt%), hectorite-2C18-2V (3 wt%).

**Figure 10:**
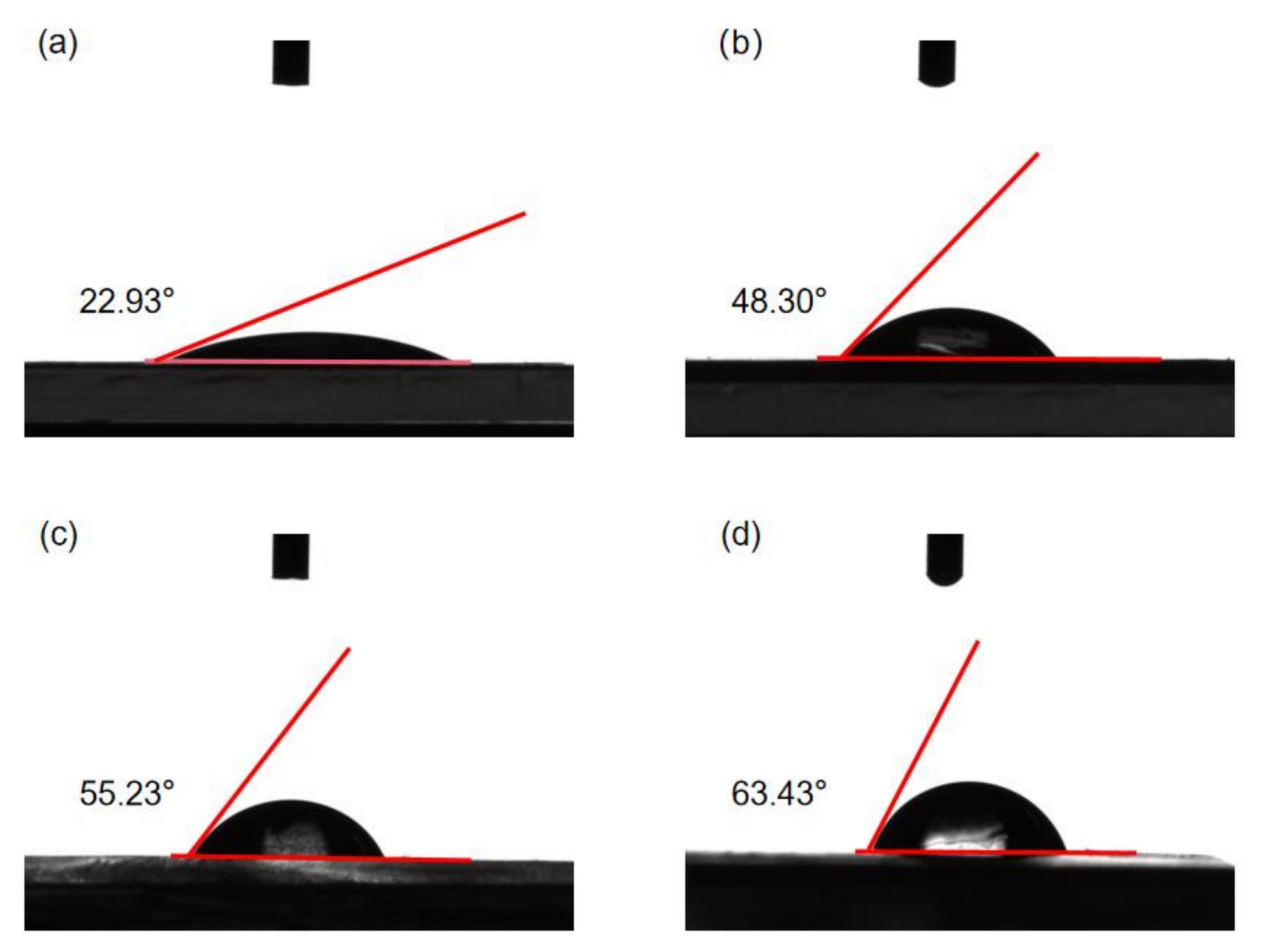
Photographs of contact angle on polyacrylate latex films prepared with (**a**) SDS (1 wt%), (**b**) Laponte-2C18 (3 wt%), (**c**) hectorite-2C18-V (3 wt%), (**d**) hectorite-2C18-2V (3 wt%).

Water absorption and water contact angles was measured to evaluate the water resistance property of the film. As shown in Figure 9, water-absorption curves of the films leveled off after 10 h of soaking; it can be judged that their water absorption was saturated and the water absorption of polyacrylate latex films prepared with hectorite-2C18-V and hectorite-2C18-2V was significantly reduced. As shown in Figure 10, the surface of polyacrylate emulsion films prepared by Pickering emulsion polymerization is more hydrophobic than that of polyacrylate emulsion films prepared with conventional surfactants. Considering Figure 9 and Figure 10, it can be demonstrated that the water resistance of the polyacrylate emulsion films prepared with hectorite-2C18-2V was significantly higher than the polyacrylate latex films prepared with SDS, hectorite-2C18, or hectorite-2C18-V.

This can be explained by the following reasons: On the one hand, the higher cross-linking density and more hydrophobic surface of the film prepared with hectorite-2C18-2V made it difficult for water molecules to diffuse into the polymer molecular segments of the film. On the other hand, the high water absorption of polyacrylate latex film caused by ester group was greatly alleviated thanks to the multi-modified hectorite nano-disks that tightly surrounded the latex particles like a raincoat. Furthermore, the avoidance of small molecule surfactants solves the problem of the degradation of water resistance properties caused by the migration of small molecules.

## 4. Conclusions

Polyacrylate latexes were prepared by Pickering emulsion polymerization without the addition of conventional small molecule surfactants. Long-chain alkyl, vinyl, and siloxy groups were successfully grafted onto the edges of the hectorite by hydrolytic condensation of silanes, so that the multi-modified hectorite was not only moderately wettable but also reactive. The properties of contact angle, O/W emulsions, and polyacrylate latex indicate that proper wettability is important to stabilize emulsions and that hectorite-C18-2V has excellent emulsification properties comparable to those of conventional emulsifiers. The role of reactive groups in stabilizing emulsions and film formation was verified by studying the performance of polyacrylate latex and films. The whole study not only proved the stability of the polyacrylate latex prepared by hectorite-C18-2V, but also demonstrated the improved mechanical properties and better water resistance of Pickering polyacrylate latex coatings.

## Figures and Tables

**Table 1:** Formulation for modifying hectorite.

	Type of Modified Particle	Quantity of Modifier (wt%)	
		OTES	VTMS
1	hectorite-C18	10	0
2	hectorite-C18-V	10	10
3	hectorite-2C18	20	0
4	hectorite-2C18-V	20	10
5	hectorite-2C18-2V	20	20

**Table 2:** Mean droplet diameters of emulsions stabilized by different emulsifiers.

	Type of Modifiers	Mean Droplet Diameter of Pickering Emulsions (µm)
a	SDS	10 ± 4
b	unmodified hectorite	54 ± 10
c	hectorite-C18	50 ± 11
d	hectorite-C18-V	45 ± 15
e	hectorite-2C18	30 ± 6
f	hectorite-2C18-V	25 ± 5
g	hectorite-2C18-2V	13 ± 5

## Data Availability

The data presented in this study are available on request from the corresponding author.
